# Role of G3BP1 phosphorylation in the regulation of plant immunity in *Arabidopsis thaliana*

**DOI:** 10.3389/fpls.2026.1777344

**Published:** 2026-05-25

**Authors:** Fatimah Abdulhakim, Aala Abulfaraj, Naganand Rayapuram, Heribert Hirt

**Affiliations:** 1 Darwin21 Desert Research Initiative, Biological and Environmental Science and Engineering Division, 4700 King Abdullah University of Sciences and Technology, Thuwal, Saudi Arabia; 2 The National Agricultural Development Company (NADEC), Riyadh, Saudi Arabia; 3 Biological Sciences Department, College of Science & Arts, King Abdulaziz University, Rabigh, Saudi Arabia

**Keywords:** condensate, stress granule, RNA-binding protein, G3BP1, phosphorylation, plant immunity

## Abstract

A central regulator of condensate formation in mammals is the Ras GTPase-activating protein SH3 domain-binding protein (G3BP) family of RNA-binding proteins. In Arabidopsis, G3BP homologs can also form condensates and exhibit diverse expression patterns and subcellular localization. Previously, we identified G3BP1 as a negative regulator of plant immunity that is phosphorylated at Ser257 *in vivo*. Here, we generated phospho-mimic (G3BP1^D^) and phospho-dead (G3BP1^A^) variants and expressed them in Arabidopsis, revealing that the phosphorylation state of G3BP1 affects susceptibility to bacterial infection by influencing ROS production and salicylic acid (SA) accumulation. G3BP1 phosphorylation also influences stomatal immunity by maintaining stomatal opening, thereby modulating pre-invasive defense mechanisms. Furthermore, we show that phosphorylation at Ser257 contributes to the stabilization of G3BP1 by limiting its degradation. Collectively, these findings identify G3BP1 phosphorylation as an important regulatory mechanism in plant immunity and provide new insights into the role of RNA-binding proteins in plant defense responses.

## Introduction

Most organisms adapt to environmental stresses by reprogramming their mRNA metabolism. Stress granules (SGs) coordinate cellular mRNA metabolism with growth, development, and stress responses. They are evolutionarily conserved cytoplasmic RNA–protein storage sites that form under adverse conditions and predominantly harbor translationally inactive mRNAs. SGs disassemble and release mRNAs into a translationally active form upon stress relief. Ras GTPase-activating protein SH3 domain-binding proteins (G3BPs or Rasputins) are “scaffolds” for the assembly and stability of SGs, which coordinate receptor-mediated signal transduction with RNA metabolism (Abulfaraj et al., 2021). G3BPs are a highly conserved family of RNA-binding proteins (RBPs) found across eukaryotes (Tourriere et al., 2003) and contain four distinct domains. The NTF2-like domain, located at the N-terminus, is involved in nucleocytoplasmic transport by interacting with RanGTP at the nuclear pore (Suyama et al., 2000), although this function remains to be fully confirmed (Macara, 2001). Additionally, the NTF2-like domain mediates protein–protein interactions and facilitates dimerization of G3BP, which is important for its structural stability and function (Alam & Kennedy, 2019; Kennedy et al., 2001). The acidic and proline-rich (PxxP) region located in the central part of G3BPs facilitates protein–protein interactions, particularly by serving as a binding site for SH3 domain-containing proteins, which are key components of signal transduction pathways (Alam & Kennedy, 2019). The C-terminal region of G3BPs contains two RNA-binding domains, an RGG box and an RNA recognition motif (RRM). The RGG box is commonly found in RNA-binding proteins, enhancing RNA binding, nuclear translocation, and post-transcriptional modifications and thus linking G3BPs to RNA metabolism (Nichols et al., 2000; Darnell et al., 2001; Alam & Kennedy, 2019). The RRM interacts with RNA via a β-sheet platform and contributes to RNA stability and post-transcriptional regulation (Nagai et al., 1995; Irvine et al., 2004; Alam & Kennedy, 2019).

The Arabidopsis genome (TAIR10) encodes eight G3BP homologs that share conserved structural features (Abulfaraj et al., 2018). G3BP1 is a member of the Ras GTPase-activating protein SH3 domain-binding protein (G3BP) family, named after its identification as an SH3 domain-binding protein of RasGAP (Parker et al., 1996). Recent studies have started to explore the roles of G3BPs in plants. Several *Arabidopsis* G3BPs form granule-like structures in response to heat stress, suggesting a conserved function for G3BPs in the dynamics of stress granules (SGs) across eukaryotes (Reuper et al., 2021). Moreover, *Arabidopsis* G3BP7 (At5G43960) localizes to SGs and contributes to resistance to viruses by interacting with viral proteins (Krapp et al., 2017). We identified G3BP1 as a negative regulator of plant immunity, as G3BP1 loss-of-function mutants exhibit stomatal closure, increased expression of key defense marker genes, and enhanced resistance to Pseudomonas syringae pv. tomato (Pst) (Abulfaraj et al., 2018). Transcriptomic analyses revealed that *Arabidopsis* G3BPs are differentially regulated under diverse environmental stresses, including heat, cold, high light, and pathogen infections, suggesting that G3BPs may contribute to adaptation to multiple stresses (Abulfaraj et al., 2021).

In a phosphoproteomic study, we identified Arabidopsis G3BP1 as a phosphoprotein with a putative role in RNA metabolism (van Bentem et al., 2006). We later confirmed Ser257 to be the *in vivo* phosphorylated site within the DKFGVPAVSLPpSPK sequence (Rayapuram et al., 2021). However, the functional significance of G3BP1 phosphorylation remained unclear.

Plants have a highly developed immune system that protects them from pathogens (Dangl et al., 2013). Plant immune responses are composed of two main pathways: pattern-triggered immunity (PTI) and effector-triggered immunity (ETI) (Bigeard et al., 2015). PTI is activated when pattern recognition receptors (PRRs) detect conserved microbe-associated molecular patterns (MAMPs/PAMPs), such as bacterial flagellin and fungal chitin (Bigeard et al., 2015; Jones and Dangl, 2006). However, successful pathogens secrete effector proteins into plant cells through specialized secretion systems, such as the type III secretion system (T3SS), to suppress PTI and induce effector-triggered susceptibility (ETS) (Macho and Zipfel, 2014; Zhang et al., 2007; Alhoraibi et al., 2019).

In response, plants have evolved intracellular nucleotide-binding leucine-rich repeat (NLR) receptors that recognize pathogen effectors and activate ETI, which often leads to localized cell death to prevent the spread of pathogens (Jones and Dangl, 2006; Dangl and Jones, 2001). A key component of PTI signaling is the activation of mitogen-activated protein kinases (MAPKs), which phosphorylate key downstream proteins to regulate plant immune responses (Rodriguez et al., 2010; Meng and Zhang, 2013; Bigeard et al., 2015; Komis et el., 2018). Plant defense against pathogens not only involves gene expression but also the regulation of mRNA metabolism by controlling mRNA stability, splicing, export, and decay. RNA-binding proteins (RBPs), miRNAs, and mRNA modifications such as N6-methyladenosine (m6A) are involved in fine-tuning defense responses at the post-transcriptional level.

SGs are cytoplasmic aggregates of proteins and untranslated mRNAs formed in response to stresses such as viral infections, heat, oxidation, and starvation, as a result of translational repression. Formation of SGs results from the activation of one of the eIF2 kinases by oxidative stress, heat stress, or nutrient deficiency, leading to the phosphorylation of the α-subunit of eIF2 and thereby blocking translation by accumulating initiation complexes around the transcripts (Krapp et al., 2017). Animal, plant, and yeast SGs contain translation initiation components and play an important role in modulating the stress translatome and proteome by selective storage of mRNAs and protection of proteins. The accumulation of translationally inactive mRNAs in SGs inhibits translation and subsequently their protein activity. Moreover, SGs can disassemble and allow rapid reactivation and release of mRNAs into a translationally active form upon stress recovery (Merret et al., 2013; Protter and Parker, 2016; Kosmacz et al., 2019).

In this work, we demonstrate that phosphorylation plays a key role in regulating G3BP1 function in plant immunity. Specifically, phosphorylation at Ser257 modulates multiple aspects of plant immune responses. Phosphorylation of G3BP1 at Ser257 promotes susceptibility to Pst infection by maintaining stomatal opening, suppressing ROS accumulation, downregulating PTI marker genes, and reducing SA accumulation. Additionally, phosphorylation enhances G3BP1 stability by preventing its degradation via the proteasome. Collectively, we uncover a phosphorylation-dependent mechanism that regulates G3BP1 function in plant immunity.

## Results

### *In vivo* and *in vitro* phosphorylation of G3BP1 at Ser257

**Figure 1:**
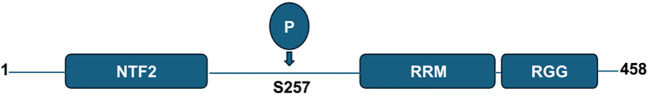
Structure and phosphorylation site of *Arabidopsis* G3BP1. N-terminal nuclear transport factor 2 (NTF2)-like domain, an acidic-rich region, a central proline-rich domain containing the Ser257 phosphorylation site (P), an RNA recognition motif (RRM), and a C-terminal arginine-glycine-rich (RGG) domain.

Several large-scale phosphoproteomic studies indicated the phosphorylation of different G3BPs by various protein kinases, including SnRK2s, TOR, and MAPKs for G3BP1 (reviewed in Abulfaraj et al., 2021). We previously identified G3BP1 as a singly phosphorylated protein at DKFGVPAVSLP(s)*PK, corresponding to Ser257 as the phosphorylated residue (Rayapuram et al., 2021; de la Fuente van Bentem et al., 2006). Besides the N-terminal NTF2 domain, which is involved in nuclear transport, and the C-terminally located RNA recognition motifs and the Gly-rich RGG domain, the central proline-rich protein–protein interaction domain contains the Ser257 phosphorylation site (**Figure 1**, Abulfaraj et al., 2021). Since the Ser257 phosphorylation motif corresponds to a MAPK consensus phosphorylation motif, we tested whether the three prominent immune MAPKs can phosphorylate G3BP1 *in vitro*. MPK3, MPK4, and MPK6 phosphorylated recombinant G3BP1 (**Supplementary Figure 1**).

Analysis by LC–MS/MS revealed a single site-specific phosphorylation at Ser257 of G3BP1 by all three MAPKs (**Supplementary Figure 1**). Although the three MAPKs phosphorylated the same Ser257 residue *in vitro* that we previously identified *in vivo*, phosphorylation changes at this site following flg22 treatment or in a MAPK-dependent manner could not be statistically validated (Rayapuram et al., 2021). We therefore cannot exclude the possibility that additional protein kinases contribute to phosphorylation of Ser257 in G3BP1. Identifying the physiological kinase(s) responsible for this modification *in vivo* will require further investigation, including analysis of MAPK mutant lines and other candidate kinases.

### Phosphorylation of G3BP1 does not alter plant morphology or subcellular localization

**Figure 2:**
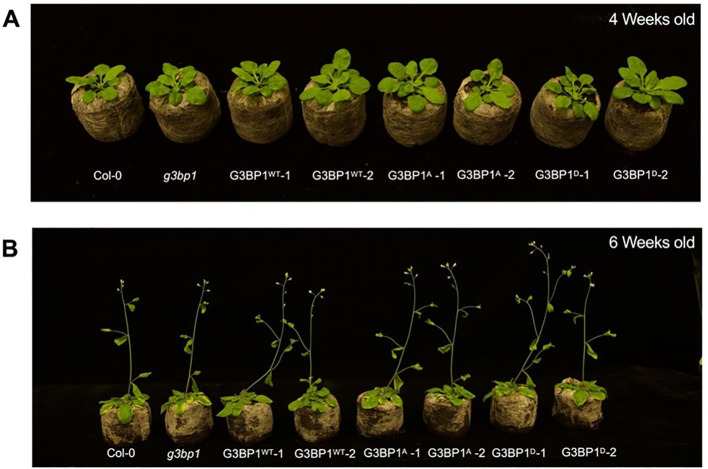
G3BP1 phosphorylation does not alter plant morphology. **(A)** Representative images of 4-week-old Col-0, *g3bp1*, G3BP1^WT^, G3BP1^A^, and G3BP1^D^. **(B)** Representative images of the same genotypes at 6 weeks, showing comparable shoot development and bolting across all lines, indicating that phosphorylation of G3BP1 does not affect plant growth and morphology overall. To determine whether phosphorylation of G3BP1 affects its subcellular localization, confocal microscopy was performed on 5-day-old roots stably expressing G3BP1-GFP fusion proteins. GFP fluorescence was predominantly observed in the cytoplasm across all genotypes, with no differences in the patterns of localization observed between G3BP1^WT^, G3BP1^A^, and G3BP1^D^ (**Supplementary Figure 2**).

To examine the role of G3BP1 phosphorylation in Arabidopsis, we generated mutant constructs of G3BP1 by replacing Ser257 with either alanine (S257A; G3BP1^A^) to create a phospho-dead variant or with aspartic acid (S257D; G3BP1^D^) to generate a phosphor mimic variant. We then analyzed the growth of Col-0, *g3bp1*, G3BP1^WT^, G3BP1^A^, and G3BP1^D^ lines expressed under the constitutive ubiquitin promoter in the g3bp1 genetic Background. Multiple independent transgenic lines were generated for each construct and initially screened. Representative lines were selected for further analysis based on stable transgene expression and consistent phenotypic behavior across independent experiments, ensuring reproducibility of the observed phenotypes. The G3BP1^WT^-GFP complementation line restored the immune phenotypes of the *g3bp1* mutant, indicating that the GFP-tagged protein is functional *in planta*. At 4 weeks, all genotypes displayed similar rosette size and leaf morphology (**Figure 2A**). At 6 weeks, stem elongation and inflorescence development were comparable across all lines (**Figure 2B**), indicating that phosphorylation of G3BP1 does not significantly affect plant growth or morphology. Confocal laser scanning microscopy of 5-day-old *Arabidopsis thaliana* roots stably expressing G3BP1^WT^-GFP, G3BP1^A^-GFP, and G3BP1^D^-GFP revealed no major differences in subcellular localization (**Supplementary Figure 2**).

### Phosphorylation of G3BP1 affects plant resistance to bacterial infection and early immune responses

**Figure 3:**
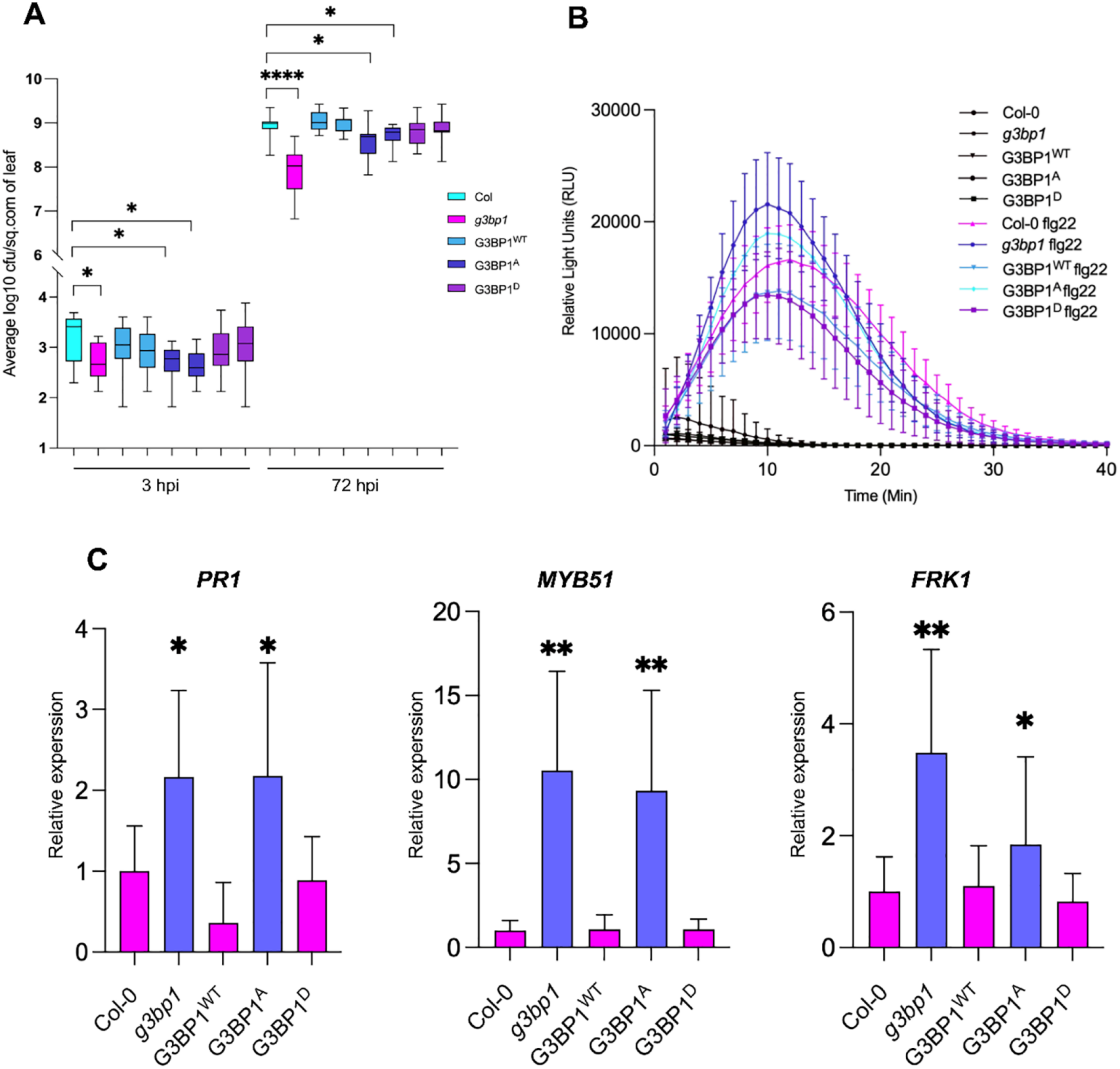
G3BP1 Phosphorylation regulates plant resistance to bacterial infection and modulates early immune responses in *Arabidopsis.* **(A)** Bacterial titers of *Pseudomonas syringae* pv*. tomato* DC3000 (Pst DC3000) quantified at 3 and 72 h post-inoculation (hpi) in Col-0, *g3bp1*, G3BP1^WT^, G3BP1^A^, and G3BP1^D^. The *g3bp1* and G3BP1^A^ plants exhibited reduced bacterial titers compared with Col-0, whereas G3BP1^D^ showed similar susceptibility to Col-0. Data represent mean ± SD from three independent biological experiments. Statistical significance was determined using the Mann–Whitney U test (P ≤ 0.01). **(B)** Reactive oxygen species (ROS) production measured as relative light units (RLU) in response to 1 µM flg22 treatment. Leaf discs from Col-0, *g3bp1*, G3BP1^WT^, G3BP1^A^, and G3BP1^D^ were assessed using a luminol-based assay over 40 minutes. The *g3bp1* and G3BP1^A^ plants exhibited significantly enhanced ROS accumulation compared with Col-0 and G3BP1^D^. Data represent mean ± SD from 12 leaf discs per genotype. **(C)** Expression levels of pattern-triggered immunity (PTI) marker genes (*PR1, MYB51*, and *FRK1*) in Col-0, *g3bp1*, G3BP1^WT^, G3BP1^A^, and G3BP1^D^ under mock conditions. Transcript levels were quantified by qRT-PCR, normalized to *UBQ10* and *ACTIN*, and expressed relative to Col-0 (set to 1). Error bars represent mean ± SD from three independent biological experiments. Statistical significance was determined using one-way ANOVA followed by Tukey’s multiple comparisons test (*P ≤ 0.05, **P ≤ 0.01, ***P ≤ 0.001).

To examine the role of G3BP1 phosphorylation in plant immunity, pathogen assays were performed using *Pseudomonas syringae* pv*. tomato* DC3000 (Pst DC3000) in Col-0, *g3bp1*, G3BP1^WT^, G3BP1^A^, or G3BP1^D^. At 3 h post-infection (hpi), the *g3bp1* mutants and G3BP1^A^ lines exhibited significantly lower bacterial titers compared with Col-0, whereas G3BP1^WT^ and G3BP1^D^ plants showed bacterial titers comparable to Col-0 (**Figure 3A**). At 72 hpi, bacterial titers were similarly high in Col-0, G3BP1^WT^, and G3BP1^D^ plants, whereas G3BP1^A^ plants showed partial resistance and the *g3bp1* mutant displayed strong resistance to pathogen infection (**Figure 3A**).

To assess the effects of phosphorylation of G3BP1 on early pathogen-associated molecular pattern (PAMP)-triggered immunity (PTI) responses, reactive oxygen species (ROS) production was measured in leaf discs under mock conditions and after treatment with 1 μM flg22. Upon flg22 treatment, *g3bp1* mutants and G3BP1^A^ plants exhibited significantly higher ROS bursts, resulting in increased ROS levels compared with Col-0, G3BP1^WT^, and G3BP1^D^ plants. In contrast, G3BP1^WT^ and G3BP1^D^ plants displayed comparable ROS production levels to Col-0 (**Figure 3B**).

To further investigate the role of G3BP1 phosphorylation in immune signaling, the transcript levels of pattern-triggered immunity (PTI) marker genes were analyzed in 14-day-old seedlings under normal growth conditions. The expression levels of Pathogenesis-Related 1 (*PR1*), MYB Domain Protein 51 (*MYB51*), and Flg22-Induced Receptor Kinase 1 (*FRK1*) were generally higher in g3bp1 mutants and G3BP1A plants than in Col-0, G3BP1^WT^, and G3BP1^D^ plants, although the magnitude of the effect and statistical significance varied between genes (**Figure 3C**). Together, these results support the conclusion that loss of G3BP1 phosphorylation is associated with enhanced expression of defense-related genes, whereas the phospho-mimic G3BP1D behaved similarly to Col-0 plants.

### Phosphorylation of G3BP1 modulates stomatal aperture in *Arabidopsis*

**Figure 4:**
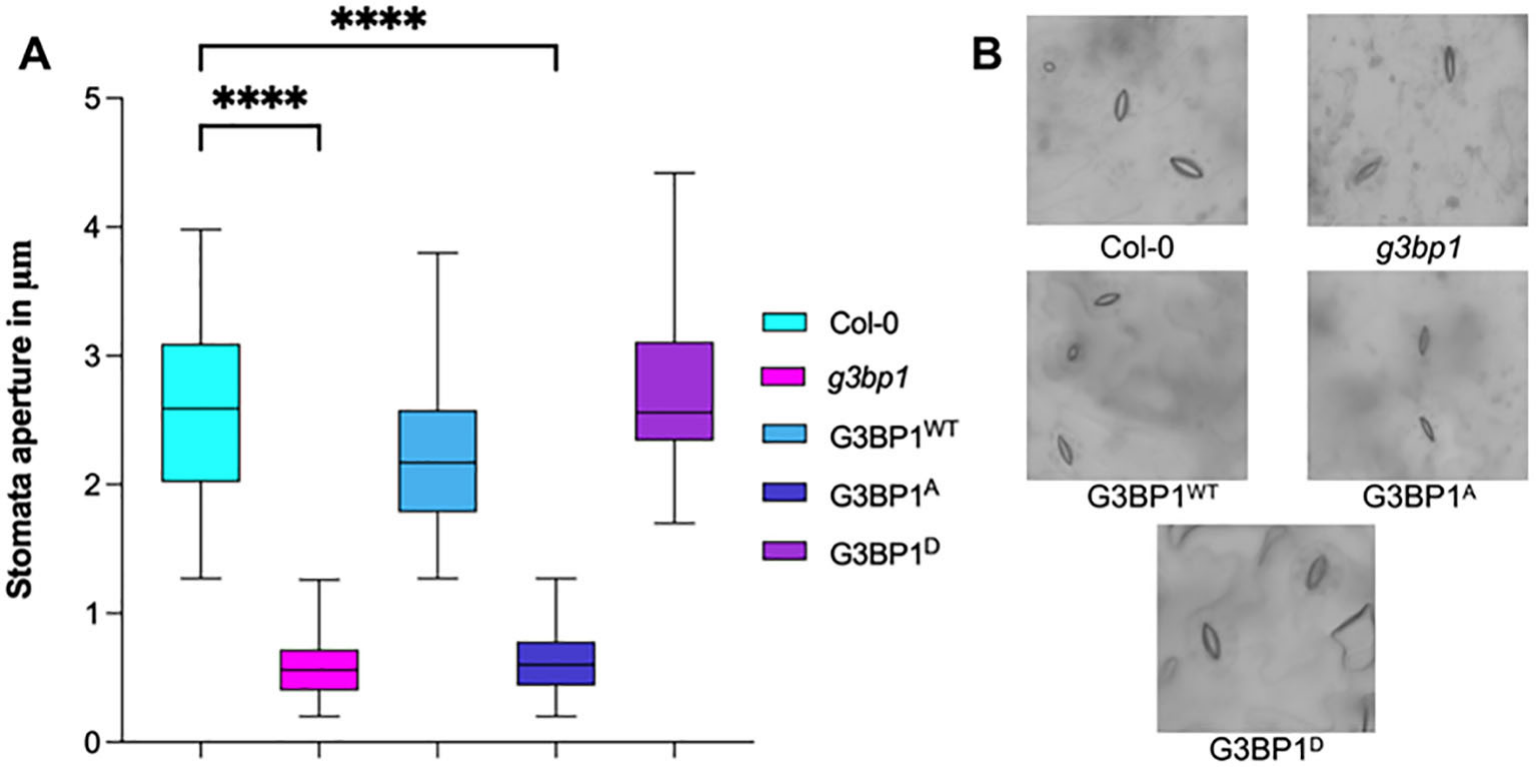
G3BP1 phosphorylation modulates stomatal aperture in *Arabidopsis*. **(A)** Stomatal aperture measurements in Col-0, *g3bp1* mutants, and stable *Arabidopsis* lines expressing G3BP1^WT^, G3BP1^A^, and G3BP1^D^. Box plots represent the median, interquartile range (IQR), and whiskers indicating the minimum and maximum values. Statistical significance was assessed using one-way ANOVA followed by Tukey’s multiple comparisons test (***P ≤ 0.001). **(B)** Representative images of stomatal morphology under mock conditions in Col-0, g3bp1, G3BP1^WT^, G3BP1^A^, and G3BP1^D^ plants.

To investigate the role of G3BP1 phosphorylation in stomatal regulation, stomatal aperture measurements were performed in Col-0*, g3bp1* mutants, and stable *Arabidopsis* lines expressing G3BP1^WT^, G3BP1^A^, and G3BP1^D^. Stomatal aperture measurements revealed significant differences between the genotypes (**Figure 4A**). Under mock conditions, the *g3bp1* mutants and G3BP1^A^ lines exhibited significantly smaller stomatal apertures (median apertures of 0.75 µm and 1.0 µm, respectively). In contrast, Col-0 and G3BP1^WT^ plants displayed moderately open stomata (2.5 µm and 2.0 µm, respectively), whereas G3BP1^D^ plants showed stomatal apertures comparable to those observed in Col-0 (~3.0 µm). Representative images of stomatal morphology further illustrate the differences in stomatal behavior among genotypes (**Figure 4B**). Col-0 and G3BP1^WT^ plants exhibited moderately open stomata under mock conditions, whereas *g3bp1* and G3BP1^A^ lines showed nearly closed stomata. G3BP1^D^ plants displayed stomatal apertures similar to those observed in Col-0, consistent with the quantitative measurements. Overall, these results suggest that phosphorylation of G3BP1 contributes to the regulation of stomatal aperture, whereas loss of phosphorylation leads to constitutive stomatal closure, highlighting its role in modulating pre-invasive immunity.

### Phosphorylation of G3BP1 regulates stomatal immunity via SA signaling

**Figure 5:**
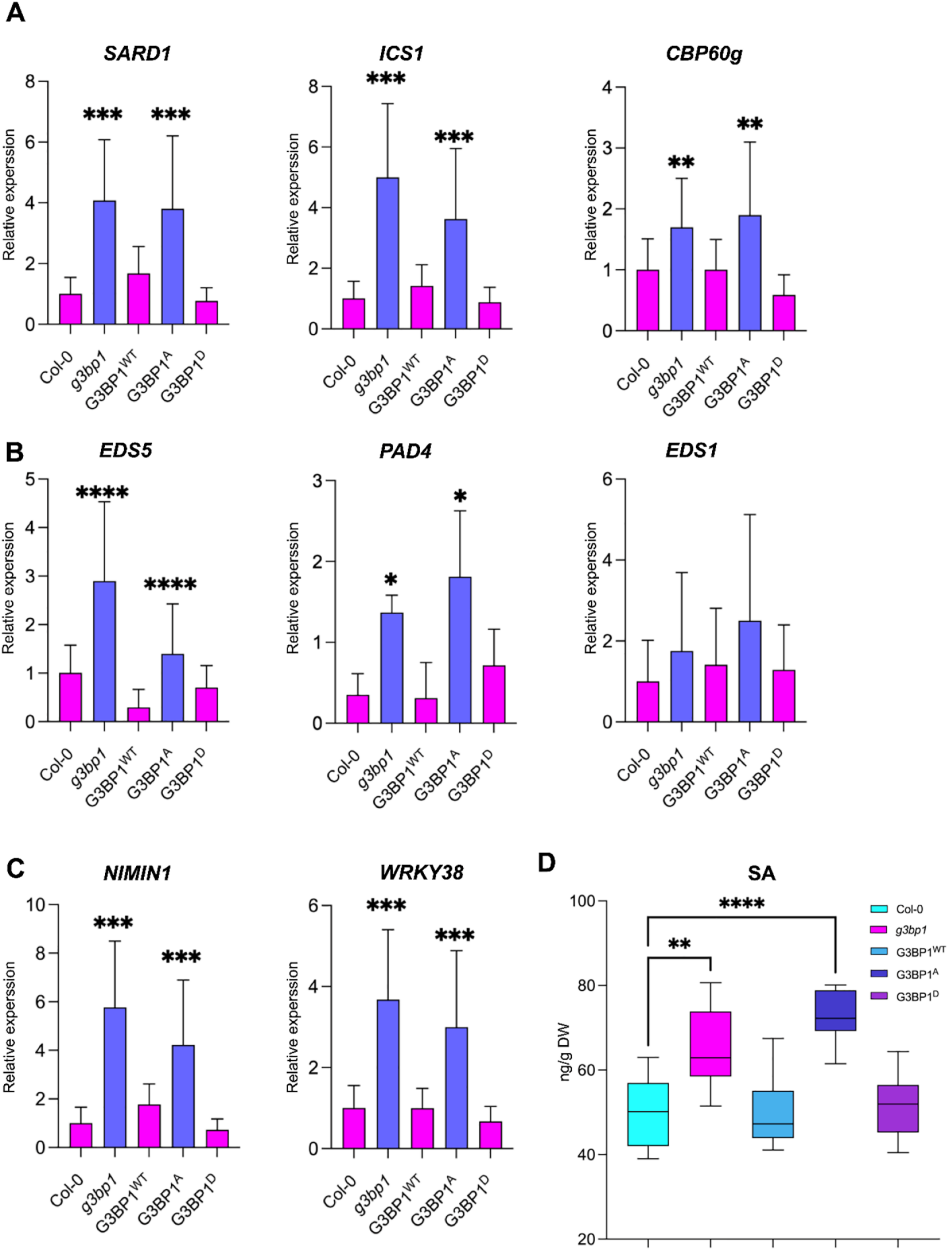
Endogenous accumulation of salicylic acid (SA) in *g3bp1* and phospho-dead G3BP1A mutants reflects altered SA-related gene expression and SA accumulation. **(A)** Expression of SA biosynthesis-related genes (*SARD1, ICS1, CBP60g)* in Col-0, *g3bp1*, G3BP1^WT^, G3BP1^A^ (phospho-dead), and G3BP1^D^ (phospho-mimic) plants. **(B)** Expression of SA accumulation and signaling-related genes (*EDS5, PAD4, EDS1*). **(C)** Expression of SA signaling-related genes (*NIMIN1, WRKY38*). Transcript levels were quantified by qRT-PCR, normalized to *UBQ10* and *ACTIN*, and expressed relative to Col-0 (set to 1). Error bars represent mean ± SD from three independent biological experiments. Statistical significance was determined using one-way ANOVA followed by Tukey’s multiple comparisons test (*P ≤ 0.05, **P ≤ 0.01, ***P ≤ 0.001, ****P ≤ 0.0001). **(D)** Endogenous SA levels in 4-week-old Col-0, *g3bp1*, G3BP1^WT^, G3BP1^A^, and G3BP1^D^ plants. Data are shown as box plots with median values, and statistical significance was determined using one-way ANOVA followed by Tukey’s multiple comparisons test.

To investigate the role of G3BP1 phosphorylation in salicylic acid (SA)-mediated defense pathways, we quantified the expression levels of genes involved in SA biosynthesis, accumulation, and signaling in Col-0, *g3bp1* mutants, and stable *Arabidopsis* lines expressing G3BP1^WT^, G3BP1^A^, and G3BP1^D^. Quantitative RT-PCR analysis showed that SA biosynthesis-related genes, including SAR Deficient 1 (*SARD1*), Isochorismate Synthase 1 (*ICS1*), and Calmodulin-Binding Protein 60g (*CBP60g*), were generally upregulated in *g3bp1* and G3BP1^A^ plants compared with Col-0, G3BP1^WT^, and G3BP1^D^ lines, although the magnitude and statistical significance varied among genes (**Figure 5A**).

Similarly, genes associated with SA accumulation and signaling, including Enhanced Disease Susceptibility 5 (*EDS5*), Phytoalexin Deficient 4 (*PAD4*), and Enhanced Disease Susceptibility 1 (*EDS1*), showed a tendency toward increased expression in *g3bp1* and G3BP1^A^ backgrounds compared to the other genotypes (**Figure 5B**). In addition, the SA signaling regulators NIM1-Interacting 1 (*NIMIN1*) and WRKY DNA-Binding Protein 38 (*WRKY38*) were upregulated in *g3bp1* and G3BP1A plants, with WRKY38 showing a pronounced increase relative to Col-0, G3BP1^WT^, and G3BP1^D^ (**Figure 5C**).

To further assess the impact of G3BP1 phosphorylation on SA accumulation, endogenous SA levels were measured in 4-week-old plants (**Figure 5D**). In line with these observations, SA levels were elevated in *g3bp1* and G3BP1A plants compared with Col-0, G3BP1^WT^, and G3BP1^D^. Together, these results indicate that loss of G3BP1 phosphorylation is associated with enhanced SA-related defense responses, whereas the phospho-mimic G3BP1D behaves similarly to wild-type plants.

### Phosphorylation regulates the stability of G3BP1 protein

**Figure 6:**
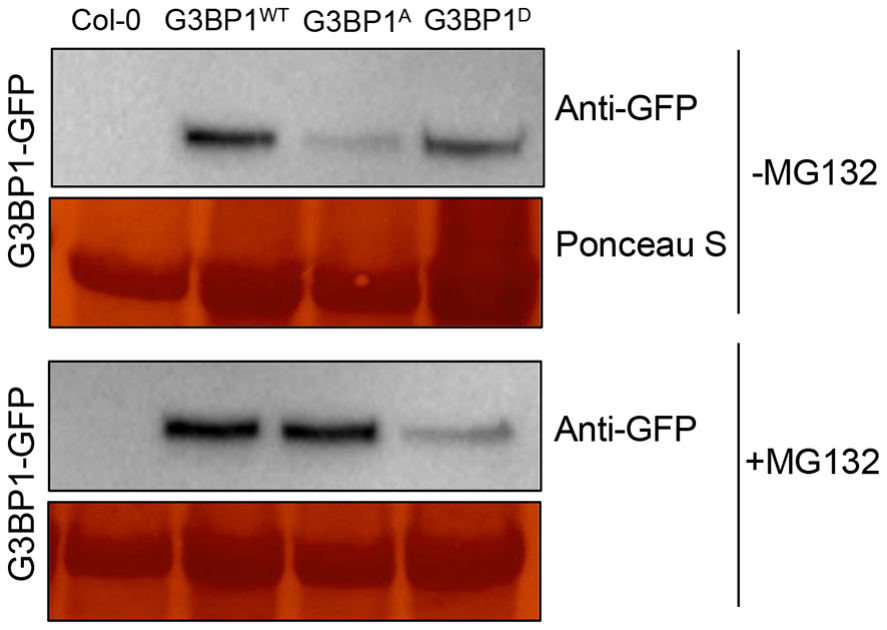
Phosphorylation at Ser257 affects G3BP1 protein stability. Immunoblot analysis of G3BP1-GFP in stable *Arabidopsis* lines expressing G3BP1^WT^, G3BP1^A^ (phospho-dead), or G3BP1^D^ (phospho-mimic). Col-0 was included as a negative control. Total protein was extracted from plants treated with or without 50 μM MG132 for 2 h. G3BP1-GFP protein levels were detected by immunoblotting using an anti-GFP antibody, and Ponceau S staining was used as a loading control.

To determine the impact of G3BP1 phosphorylation on protein stability, we analyzed the levels of G3BP1-GFP fusion proteins in stable *Arabidopsis* lines expressing G3BP1^WT^, G3BP1^A^, and G3BP1^D^ using immunoblotting. Col-0 was included as a negative control. Total protein was extracted from plants treated with or without the proteasome inhibitor MG132. In the absence of MG132, G3BP1^A^-GFP protein levels were markedly reduced compared with G3BP1^WT^, indicating reduced stability of the phospho-dead G3BP1^A^ protein (**Figure 6**, top panel).

To determine whether this reduction results from proteasome-dependent degradation, plants were treated with 50 μM MG132 for 2 h prior to protein extraction and immunoblot analysis. MG132 treatment restored G3BP1A-GFP protein levels in plants expressing G3BP1^A^-GFP to levels comparable to G3BP1^WT^ and G3BP1^D^ (**Figure 6**, bottom panel), indicating that the phospho-dead G3BP1^A^ protein undergoes proteasome-dependent degradation. These results suggest that phosphorylation at Ser257 contributes to G3BP1 stabilization by reducing its susceptibility to proteasome-mediated degradation.

## Discussion

G3BPs play a central role in the regulation of mRNA metabolism, including translational control and mRNA stability, thereby contributing to cellular homeostasis and gene expression at the post-transcriptional level (Abulfaraj et al., 2021). Here, we provide evidence that G3BP1 is phosphorylated and that its phosphorylation influences immune responses by promoting susceptibility to bacterial infection, suppressing ROS accumulation, and modulating SA-related defense pathways. Previous studies have characterized the localization and functional diversity of the *Arabidopsis* G3BP family, which comprises eight members, several of which localize to stress granules (SGs) under various stress conditions (Reuper et al., 2021). Given the central role of G3BPs in stress granule assembly, our findings suggest that G3BP1 phosphorylation regulates immune responses through post-transcriptional mechanisms. Notably, phosphorylation affects stomatal immunity, ROS production, and SA signaling, indicating that G3BP1 integrates multiple layers of plant defense. However, because these immune phenotypes are not directly linked to stress granule formation, G3BP1 function in immunity may occur independently of classical stress granule dynamics. A detailed subcellular localization analysis of seven AtG3BP family members demonstrated that all localize to the cytoplasm under normal conditions, with AtG3BP4 also showing nuclear localization. In addition, several AtG3BPs, including AtG3BP6, AtG3BP2, and AtG3BP1, form granule-like structures even under non-stress conditions, while all members form such structures upon heat stress (Reuper et al., 2021). Functionally, only a subset of AtG3BPs has been characterized in plants. AtG3BP7 has been implicated in viral immunity (Krapp et al., 2017), whereas AtG3BP1 has been identified as a negative regulator of bacterial immunity (Abulfaraj et al., 2018).

The differential expression of AtG3BP family members across developmental stages, tissues, and stress conditions suggests that they may participate in diverse biological processes (Abulfaraj et al., 2021). Proteomic analyses have identified numerous SG-resident proteins in *Arabidopsis*, including RNA-binding proteins, translation regulators, and protein kinases involved in growth and stress responses (Gutierrez-Beltran et al., 2015; Kosmacz et al., 2019). Notably, components of the N6-methyladenosine (m6A) RNA modification machinery are enriched in SGs in both plants and animals (Kosmacz et al., 2019). In mammalian systems, G3BPs are negatively affected by m6A modification (Edupuganti et al., 2017; Zhao et al., 2017), and m6A plays a major role in regulating mRNA fate, including stability, splicing, and translation (Wang et al., 2014; Zhao et al., 2014; Wang et al., 2015). G3BP1 contributes to mRNA stability through multiple mechanisms, including binding to target mRNAs and protecting them from degradation. Although these mechanisms are well established in animal systems, it remains unclear whether plant.

G3BPs function similarly. Given the involvement of both m6A machinery and AtG3BP1 in plant immunity, their co-localization in SGs suggests a potentially coordinated regulatory mechanism that warrants further investigation. Although not directly tested in this study, this potential interaction highlights an additional layer of post-transcriptional regulation that could contribute to immune responses. Overall, our study establishes that phosphorylation of G3BP1 regulates multiple facets of plant immunity. These findings highlight the functional importance of the Ser257 phosphorylation site and reveal a mechanism linking protein phosphorylation to post-transcriptional control of immune responses in plants. Although this residue can be phosphorylated by MAPKs *in vitro*, we did not observe significant changes in MAPK mutants, and thus cannot establish a direct role for MAPKs in mediating G3BP1 phosphorylation *in planta*. This suggests that additional protein kinases beyond MAPKs are likely to be responsible for targeting G3BP1 *in vivo*. Consistent with these observations, our findings suggest that G3BP1 represents a negative regulator of plant immunity, with Ser257 phosphorylation acting as a key regulatory site. Phosphorylation at Ser257 promotes bacterial susceptibility, suppresses ROS accumulation, influences SA-associated defense gene expression as well as overall SA accumulation, and regulates stomatal aperture, thereby positioning AtG3BP1 as an important regulator of plant immune responses. These results further indicate that G3BP1 phosphorylation influences both pre-invasive defenses, such as stomatal regulation, and post-invasive immune responses, including ROS production and defense-related gene expression. This is consistent with previous findings demonstrating that AtG3BP1 negatively regulates SA-dependent immunity (Abulfaraj et al., 2018), and our results further show that these responses are strongly influenced by its phosphorylation status.

Given the central role of G3BPs in RNA metabolism and stress granule dynamics, an important next step will be to determine how G3BP1 phosphorylation regulates RNA metabolism and mRNA fate, and whether these functions operate independently of stress granules. Furthermore, identifying the upstream kinases responsible for G3BP1 phosphorylation will be critical for understanding how immune signaling pathways converge on post-transcriptional regulatory mechanisms. Together, these findings position G3BP1 phosphorylation as a key regulatory node linking post-transcriptional control to multiple layers of plant immune responses.

## Materials and methods

### Plant materials and growth conditions

*Arabidopsis thaliana* ecotype Columbia (Col-0) (NASC ID: N1092) was used as the wild-type plant material, along with the T-DNA insertion mutant g3bp1 (SAIL_1153_H01) obtained from the Nottingham *Arabidopsis* Seed Centre (NASC).Seeds were surface sterilized by vortexing in 1 mL of 70% ethanol containing 0.01% Triton X-100 for 10 min at room temperature, followed by three washes with absolute ethanol. The seeds were then dried on sterile Whatman paper in a laminar flow hood and stratified at 4 °C on half-strength Murashige and Skoog (MS) medium for at least 2 days.

The 1/2 MS medium contained Murashige and Skoog basal salts with minimal organics, 0.05% MES hydrate, and 0.5% agar (Sigma A4675, St. Louis, MO, USA), adjusted to pH 5.7 with KOH. Seedlings were grown in a growth chamber under controlled environmental conditions of 23 °C (day)/22 °C (night) with a 16 h light/8 h dark photoperiod. For long-term growth, plants were transferred to Jiffy pots and maintained in Percival growth chambers under short-day conditions (8 h light/16 h dark, 60% humidity) at 23 °C (day)/22 °C (night) for four weeks.

### *In vitro* kinase assays

*In vitro* kinase assays were performed in a 20 μL reaction volume containing purified recombinant G3BP1 protein and constitutively active MAPKs at a 1:10 kinase-to-substrate ratio, along with 20 mM Tris-HCl (pH 7.5), 10 mM MgCl_2_, 5 mM EGTA, 1mM DTT, and 50 μM ATP. Reactions were incubated at room temperature (RT) for 30 minutes and terminated by the addition of SDS sample buffer, followed by denaturation at 95 °C for 10 minutes. Proteins were resolved by SDS-PAGE and visualized using SimplyBlue™ SafeStain (Novex, Cat. No. LC6065). Protein bands of interest were excised, destained using acetonitrile (ACN) and 100 mM ammonium bicarbonate (NH_4_HCO_3_) (four washes, 15 min each), and subjected to reduction with 10 mM Tris(2-carboxyethyl) phosphine (TCEP, Sigma, Cat. No. C-4706) at 37 °C for 1 hour. Alkylation was performed using 20 mM S-methyl methanethiosulfonate (MMTS, Sigma, Cat. No. 64306) at RT for 30 minutes. Gel pieces were digested overnight at 37 °C with porcine trypsin (Promega) and peptide extraction was performed using ACN and 1% formic acid. Peptides were desalted using C18 ZipTip^®^ columns (Millipore, Cat. No. ZTC18S096) and analyzed by LC-MS/MS. Data were processed using the Mascot server and searched against the TAIR10 database to identify phosphorylation sites.

### Generation of phosphorylation lines: site-directed mutagenesis and generation of phospho-mimic and phospho-dead mutants

Site-directed mutagenesis of G3BP1 cDNA was employed to generate phospho-dead and phospho-mimic versions of G3BP1. PCR reactions were performed using Phusion High-Fidelity DNA Polymerase (New England Biolabs) and primers designed to introduce specific mutations at serine residues (e.g., S-to-A for phospho-dead and S-to-D for phospho-mimic variants). A DNA plasmid template (5–20 ng) was used as the starting material for each reaction. Post-PCR, the DpnI restriction enzyme (Promega) was utilized to selectively digest the parental methylated DNA. The digestion mixture, consisting of 5 μL of purified PCR product and 1 μL of DpnI (10 U/μL), was incubated at 37 °C for 1 hour. The digested products were then transformed into E. coli by heat-shock transformation. Transformants were screened on selective media, and colonies were picked for sequencing analysis to confirm the introduction of the desired mutations. Confirmed plasmid constructs containing the phospho-dead (S-to-A) and phospho-mimic (S-to-D) mutations were subsequently cloned into pAA9(B1), containing the ubiquitin promoter and recombined into pGWB433 vector by LR reaction following the manufacturer’s instructions to obtain pAA(B2). The constructs were confirmed by sequencing and used to stably transform the *g3bp1* T-DNA insertion line via floral dip transformation using Agrobacterium tumefaciens C58. Transgenic plants were selected on 1/2 MS medium containing 50 μM kanamycin. Lines expressing the G3BP1 wild type, phospho-dead, and phospho-mimic variants were identified through three generations of selection and used for subsequent experiments.

### Pathogen assays

*Arabidopsis* plants (Col-0, *g3bp1* mutants, and lines expressing G3BP1^WT^, G3BP1^A^, and G3BP1^D^) were grown for four weeks under short-day conditions (8 hours light/16 hours dark, 22 °C). *Pseudomonas syringae* pv. *tomato* DC3000 (Pst DC3000) cultures were prepared by streaking glycerol stock onto NYGA plates supplemented with rifampicin (50 mg/L) and incubation at 28 °C for 48 hours. For inoculations, bacterial cultures were adjusted to OD600 = 0.2 in 10 mM MgCl_2_ containing 0.04% (v/v) Silwet L-77.

Spray inoculations were performed by spraying the bacterial suspension evenly onto the aerial parts of the plants until thoroughly wetted. The inoculated plants were covered with a plastic dome to maintain high humidity and incubated in a growth chamber under short-day conditions. Bacterial growth was assessed at 3 and 72 h post-infection (hpi). Leaf discs were collected from three leaves per plant from 10 individual plants per genotype for each biological replicate. Bacteria were extracted by grinding the leaf discs in 10 mM MgCl_2_ containing 0.04% (v/v) Silwet L-77. The bacterial homogenates were serially diluted 10-fold and 10 μL of each dilution was plated on LB agar containing rifampicin (50 mg/L). Plates were incubated at 28 °C for 48 hours, and bacterial colonies were counted to determine the colony-forming units (CFU) per cm² of leaf tissue. Data were collected from three biological replicates for each plant genotype.

### ROS burst assays

A luminol-based luminescence assay (Huang et al., 2013) was used to measure the production of reactive oxygen species (ROS) in *Arabidopsis* Col-0, *g3bp1* mutants, and lines expressing G3BP1^WT^, G3BP1^A^, and G3BP1^D^. Leaf discs (4 mm in diameter) were excised from four-week-old plants and placed adaxial side-up in 96-well plates (Thermo Fisher, Rochester, NY, USA). Each well contained 150 μL of sterile water, and the plates were incubated overnight at room temperature in the dark.

The following day, the water was replaced with 100 μL of reaction solution containing 50 μM luminol (Sigma, St. Louis, MO, USA), 10 μg/mL horseradish peroxidase (HRP; Sigma, St. Louis, MO, USA), and 1 μM flg22 as the microbe-associated molecular pattern (MAMP) elicitor. Water was used as a mock control. Luminescence was recorded using a TECAN Infinite 200 PRO microplate reader at 1-minute intervals for 40 minutes following the addition of the reaction solution. The signal integration time for each measurement was 0.5 seconds. ROS production was quantified as relative light units (RLU), and data were expressed as the mean RLU across three biological replicates, each containing 12 leaf discs per genotype.

### Measurement of stomatal aperture

To analyze stomatal responses, epidermal peels from 4-week-old *Arabidopsis* leaves were floated on stomatal opening buffer (10 mM KCl, 50 mM CaCl_2_, and 10 mM MES, pH 6.2) under light for 2 h (Desclos-Theveniau et al., 2012). The peels were then mounted on slides and microscopic images of stomata were captured using a Leica DM5000 confocal microscope. Internal stomatal aperture widths were measured using ImageJ software (Dow and Bergmann, 2014). A minimum of 50 stomata were analyzed per genotype.

### Quantitative RT-PCR analysis

Total RNA was extracted from 14-day-old *Arabidopsis* seedlings using the RNeasy Plant Mini Kit (Qiagen) according to the manufacturer’s instructions. RNA integrity was assessed with a NanoDrop spectrophotometer (Thermo Fisher Scientific), and 1 μg RNA was used for cDNA synthesis with the SuperScript™ III First-Strand Synthesis SuperMix kit (Invitrogen) and oligo (dT) primers. The cDNA was diluted 10-fold in nuclease-free water. qRT-PCR was performed using SsoAdvanced Universal SYBR Green Supermix (Bio-Rad) in a CFX96 Real-Time System (Bio-Rad). Each reaction contained 2 μL diluted cDNA, 5 μL SYBR Green Supermix, and 2 μL of a 330 nM primer mix, with technical triplicates per sample. Cycling conditions were 95 °C for 2 min, followed by 40 cycles of 95 °C for 15 s and 60 °C for 30 s. Melting curves confirmed the product specificity. The expression levels of *PR1, MYB51, FRK1, SARD1, ICS1, CBP60g, EDS5, PAD4, EDS1, NIMIN1, and WRKY38* were normalized to AT3G18780 (*ACTIN*) and AT4G05320 (*UBQ10*) and presented relative to Col-0 controls (set to 1.0). Data were analyzed using Bio-Rad CFX Manager software, with statistical significance determined in GraphPad Prism 9.

### Quantification of salicylic acid

Salicylic acid (SA) levels were quantified in leaves of 4-week-old *Arabidopsis thaliana* plants, including Col-0, *g3bp1* mutant, G3BP1^WT^ (complementation line), G3BP1^A^ (phospho-dead), and G3BP1^D^ (phospho-mimic). Phytohormone extraction and analysis were performed following the protocol described by Almeida Trapp et al. (2015). For chromatographic analysis, a Thermo Fisher TQS-Altis Triple Quadrupole Mass Spectrometer was coupled with a Thermo Scientific Vanquish MD HPLC system. Separation was achieved using an UPLC Kinetex C18 column (2.6 μm, 2.1 × 150 mm). The mobile phase consisted of water (A) and acetonitrile (B), with a flow rate of 0.5 mL/min. The gradient elution started at 25% B, increased linearly to 50% B over 2 minutes, then increased to 100% B over the next 6 minutes (by 8 min total). The solvent composition was maintained at 100% B until 11.4 minutes, followed by a rapid decrease to 20% B at 11.5 minutes. The column was re-equilibrated at 20% B until 14 minutes. The column temperature was maintained at 35 °C throughout the run.

### Protein stability assay

To assess protein stability, G3BP1-GFP levels were analyzed via immunoblotting. Total protein extracts were prepared from 4-week-old plants treated with or without 50 μM MG132 (Sigma-Aldrich) for 2 h. Samples were separated by SDS-PAGE (10%) and transferred onto PVDF membranes (Millipore). Immunoblotting was performed using an anti-GFP (1:5000, Abcam) and a HRP-conjugated secondary antibody (1:15000, Bio-Rad). Signal detection was performed using the ECL detection kit (Thermo Fisher Scientific).

### Statistical analysis

All experiments were conducted in three independent biological replicates, with each experiment containing at least three technical replicates per condition. Statistical analysis was performed using GraphPad Prism 9. Data were analyzed using one-way ANOVA followed by Tukey’s multiple comparisons test, unless otherwise indicated in the corresponding figure legends.

## Data Availability

Publicly available datasets were analyzed in this study. This data can be found here: Rayapuram et al. (2021). Chromatin phosphoproteomics unravels a function for AT-hook motif nuclear localized protein AHL13 in PAMP-triggered immunity. Proceedings of the National Academy of Sciences of the United States of America, 118(3), e2004670118.

## Supplementary material

Supplementary Figure 1 G3BP1 is phosphorylated by MPK3, MPK4, and MPK6 *in vitro.* (A) Coomassie blue-stained SDS-PAGE gel of the purification steps of His6-MBP- G3BP1. Lane M: molecular weight marker; Lane P: pellet fraction; Lane S: soluble fraction; Lane NR: non-retained fraction; Lanes W1 and W2: wash fractions; Lanes E2, E4, E6, E8, E10: eluted fractions; Lane B: proteins bound to Ni-NTA beads. The arrow indicates the purified His6-MBP-AtG3BP1 protein (~93 kDa). (B) Coomassie blue-stained SDS-PAGE gel showing His6-MBP-AtG3BP1 protein after desalting. The arrow indicates purified G3BP1 protein (~93 kDa). (C, E, G) SimplyBlue™ SafeStain SDS-PAGE of AtG3BP1 after phosphorylation by MPK3, MPK4, and MPK6, respectively. Arrows indicate the G3BP1 band excised for analysis. (D, F, H) LC-MS/MS spectra confirming phosphorylation of G3BP1 at Ser257 by MPK3, MPK4, and MPK6, respectively. Spectra depict peptide fragmentation with annotated b- and y-ions confirming the phosphorylation site. Mascot and Mascot Delta (MD) scores validate the phosphorylation event for MPK3 (79.6, 36.6), MPK4 (77, 35.8), and MPK6 (72, 25.2), respectively.

Supplementary Figure 2 Subcellular localization of G3BP1^WT^-GFP, G3BP1^A^-GFP (phospho-dead), and G3BP1^D^-GFP (phospho-mimic) in *Arabidopsis* root epidermal cells. Confocal laser scanning microscopy images of 5-day-old *Arabidopsis thaliana* roots stably expressing G3BP1^WT^-GFP, G3BP1^A^-GFP (phospho-dead), and G3BP1^D^-GFP (phospho-mimic). GFP fluorescence (green) indicates the localization of G3BP1 fusion proteins, while propidium iodide (PI) staining (magenta) marks the cell walls. Scale bar: 10 µm.
