# Supplementary material for: Role of G3BP1 phosphorylation in the regulation of plant immunity in Arabidopsis thaliana
Source: Front Plant Sci. 2026 May 25;17:1777344. doi: 10.3389/fpls.2026.1777344 (PMC13243370; doi:10.3389/fpls.2026.1777344)
Supplement: Supplementary Figure 1 — G3BP1 is phosphorylated by MPK3, MPK4, and MPK6 in vitro. (A) Coomassie blue-stained SDS-PAGE gel of the purification steps of His6-MBP- G3BP1. Lane M: molecular weight marker; Lane P: pellet fraction; Lane S: soluble fraction; Lane NR: non-retained fraction; Lanes W1 and W2: wash fractions; Lanes E2, E4, E6, E8, E10: eluted fractions; Lane B: proteins bound to Ni-NTA beads. The arrow indicates the purified His6-MBP-AtG3BP1 protein (~93 kDa). (B) Coomassie blue-stained SDS-PAGE gel showing His6-MBP-AtG3BP1 protein after desalting. The arrow indicates purified G3BP1 protein (~93 kDa). (C, E, G) SimplyBlue™ SafeStain SDS-PAGE of AtG3BP1 after phosphorylation by MPK3, MPK4, and MPK6, respectively. Arrows indicate the G3BP1 band excised for analysis. (D, F, H) LC-MS/MS spectra confirming phosphorylation of G3BP1 at Ser257 by MPK3, MPK4, and MPK6, respectively. Spectra depict peptide fragmentation with annotated b- and y-ions confirming the phosphorylation site. Mascot and Mascot Delta (MD) scores validate the phosphorylation event for MPK3 (79.6, 36.6), MPK4 (77, 35.8), and MPK6 (72, 25.2), respectively. [file SupplementaryFile1.docx]

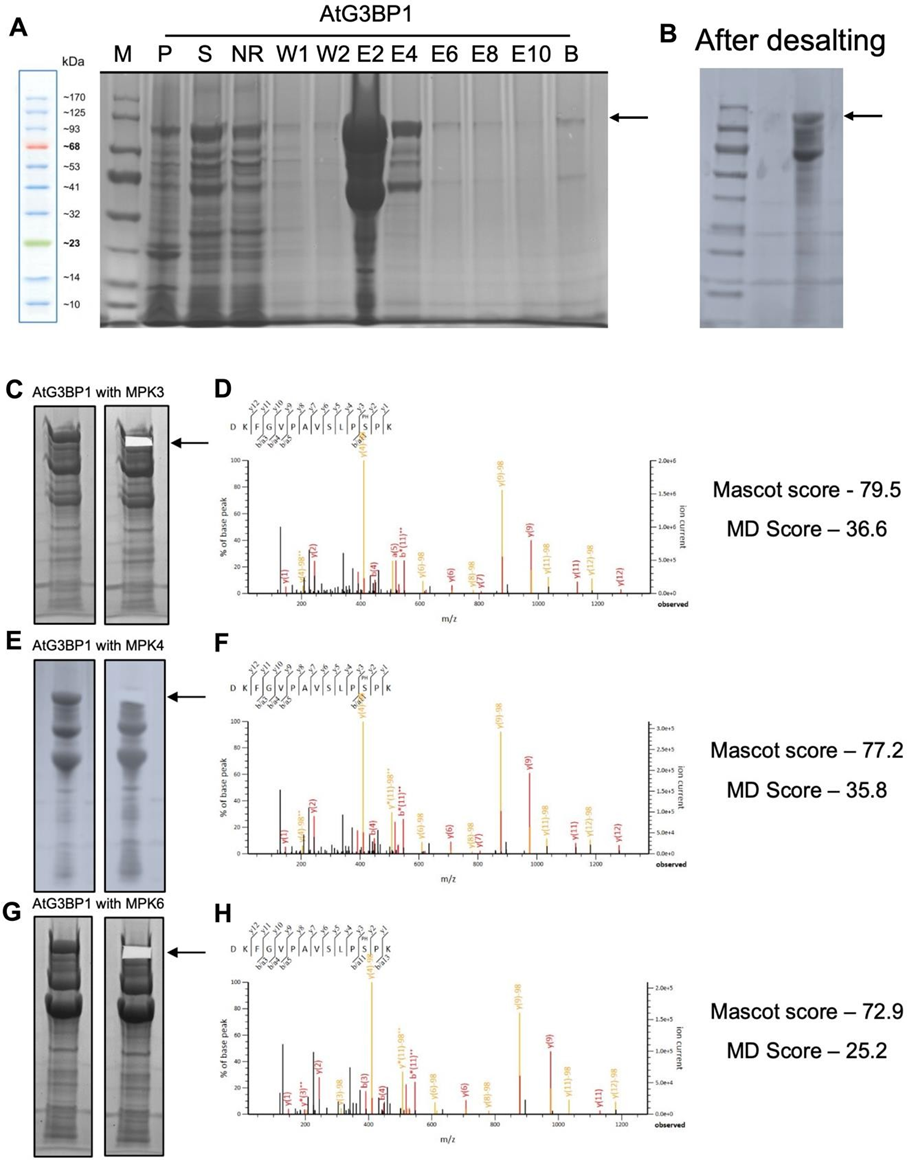


**Supplementary Figure 1. G3BP1 is phosphorylated by MPK3, MPK4, and MPK6 *in***

***vitro.* (A)** Coomassie blue-stained SDS-PAGE gel of the purification steps of His6-MBP-

G3BP1. Lane M: molecular weight marker; Lane P: pellet fraction; Lane S: soluble

fraction; Lane NR: non-retained fraction; Lanes W1 and W2: wash fractions; Lanes

E2, E4, E6, E8, E10: eluted fractions; Lane B: proteins bound to Ni-NTA beads. The arrow

indicates the purified His6-MBP-AtG3BP1 protein (~93 kDa). **(B)** Coomassie blue-stained

SDS-PAGE gel showing His6-MBP-AtG3BP1 protein after desalting. The arrow indicates

purified G3BP1 protein (~93 kDa). (C, E, G) SimplyBlue™ SafeStain SDS-PAGE of

AtG3BP1 after phosphorylation by MPK3, MPK4, and MPK6, respectively. Arrows

indicate the G3BP1 band excised for analysis. (D, F, H) LC-MS/MS spectra confirming

phosphorylation of G3BP1 at Ser257 by MPK3, MPK4, and MPK6, respectively. Spectra

depict peptide fragmentation with annotated b- and y-ions confirming the phosphorylation

site. Mascot and Mascot Delta (MD) scores validate the phosphorylation event for MPK3

(79.6, 36.6), MPK4 (77, 35.8), and MPK6 (72, 25.2), respectively.


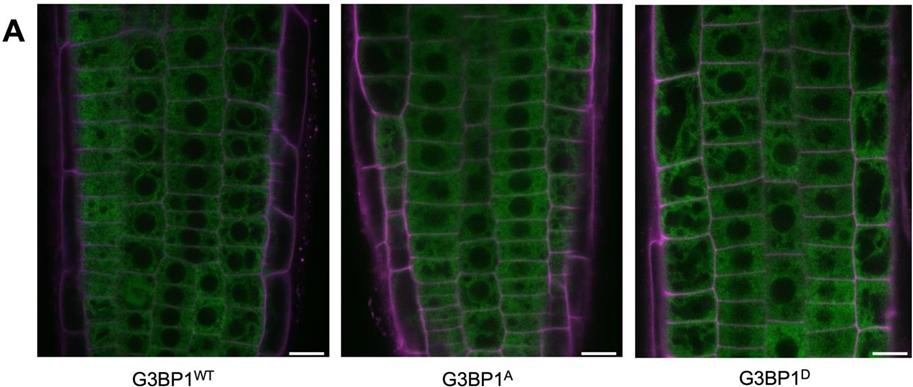


**Supplementary Figure 2. Subcellular localization of G3BP1^WT^-GFP, G3BP1^A^-GFP**

**(phospho-dead), and G3BP1^D^-GFP (phospho-mimic) in *Arabidopsis* root**

**epidermal cells.**

Confocal laser scanning microscopy images of 5-day-old *Arabidopsis thaliana* roots

stably expressing G3BP1^WT^-GFP, G3BP1^A^-GFP (phospho-dead), and G3BP1^D^-GFP

(phospho-mimic). GFP fluorescence (green) indicates the localization of G3BP1 fusion

proteins, while propidium iodide (PI) staining (magenta) marks the cell walls. Scale bar:

10 µm.
